# Editorial

**Published:** 1893-08

**Authors:** 


### Editorial.

### THE PROPER SCOPE OF MEDICAL JOURNALISM.

With a view to understand the true sphere of editorial work in connection with medical literature, it is requisite to take a cursory glance at the present status of journalism in this country and in other portions of the world.

The vast changes which have come about in the publication of various kinds, from the encyclopedia of medicine and surgery, with a large number of authors, to the medical newspaper of limited proportions, are among the most notable features of the last quarter of a century. The presentation in some extensive works of articles without the responsibility of individual authorship, is of questionable propriety, in like manner as thc appearance of articles in medical journals without the names of the writers.

The largest liberty should be allowed contributors to medical literature in the expression of their convictions and the record of their experience, as the observations of even those without scientific attainments may often prove advantageous to the profession. One of the greatest obstacles to the usefulness of medical associations and societies is the tendency in such organizations to shut out the presentation of facts by those least gifted in speech or in writing. The prominence given to those who are recognized as authorities in the different departments of practice, precludes almost entirely the appearance of the less favored class of the profession, and while there is really no sort of barrier to the expression of their views, the practical working in such bodies leads to their exclusion. It is unfortunate that the modest, retiring country doctor, should not be heard in many instances, when his original views might serve a good purpose in elucidating the question at issue.

The same attitude is occupied, to a very large extent, in the contributions to medical journals, and those familiar with the use of the pen fill up the pages of our weeklies, monthlies and quarterlies, with only rarely an article from those less known to fame, who might add materially to our stock of useful knowledge. There are some men of limited book lore whose initiative genius lies hidden from the public, who ought to have a showing in medical journals. If they are not able to write with elegance, or even gramatically, the editor may well bestow some attention upon corrections, which shall put his communication in good shape, and winnow out the chaff to secure the grain mixed with it. It should not be inferred that every member of the profession who is seized with the *cacoethes scribendi*, should be encouraged, but only those who have something valuable to present should be allowed a place in the pages of a medical journal.

There are some very learned dissertations, based entirely upon the data of others and having extensive bibliographical references, which really afford no instruction and should not be admitted to medical journals. If the studious author of such a paper thinks his researches entitled to the attention of the profession he should be willing to risk the publication of it at his own expense and not burthen the business department of a medical journal with the expense. It is not merely scientific attainment which avails in preparing an article for the readers of medical journals, but there must be practical discernment and aptitude to meet the wants of the profession. One may write most learnedly and yet give no instruction, and the practitioner looks to the medical journal for hints to guide him in the treatment of diseases. A case of some disease which presents unusual features may be reported in detail so as to show the effects of some special line of treatment and from a useful guide in the management of similar cases. It thus turns out that precedents become established by facts and we are not left to grasp our way blindly under the influence of a theory which may be misleading. True science rests upon facts, and the humblest member of the profession is capable of observing what is presented in the progress of disease and in the use of remedies, so as to aid in the accumulation of facts, which shall be subservient to an important result.

There are at the present day so many different departments of professional work that journals have sprung up to fill the demands of the various specialties, but it is still found that the claims of all can be met by embodying information of a general character, so as to furnish matter for the instruction of the different classes of practicioners in our journals.

It may not be out of place to draw attention in this connection to the mode in which the journal of the American Medical Association has been conducted latterly. This publication differs from any other in this country by virtue of its containing the papers presented in the different sections of the Association. Being of great variety, it would be eminently proper that selections should be made from each section in every number of the journal, so as to have something of note for all the members who read the contents. But there has been no such method used heretofore, and indeed there seems to be no systematic rule adopted in regard to the management, except to exclude a number of the most important papers which may have appeared in other medical journals.

The reports of societies which are published by medical journals, should be pruned of their superfluities, and long winded letters of correspondents call for abridgment. J. M'F. G.

---

The Natural Body Brace advertised in this issue of the RECORD should interest every physician, as it supplies a want which experience teaches can not possibly be obtained through any other known means.

---

We wish to call special attention to new advertisements in this issue: Rio Chemical Co., St. Louis, Mo.; Barnes Medical College, St. Louis, Mo.; Medical College of Georgia, Augusta, Ga., and the Atlanta Medical College.

---

We are a little late this month on account of moving of office. Will come out hereafter on first of each month.

